# On Diseases of the Skin

**Published:** 1857-12

**Authors:** 


*On Diseases of the Skin.* By ERASMUS WILSON, F. R. S. Fourth American, from the fourth and enlarged London edition. Blanchard & Lea. 1857.

The present edition of Wilson on the Skin, is far more complete than any of the preceding, the author having added several entire chapters and elaborated extensively, many points which have not been sufficiently prominent in the preceding editions. The new system of classification, which has now been adopted, adds materially to the usefulness of the volume. We have also an appendix of selected formulæ, consisting of prescriptions which have been found valuable in the therapeutics of skin affections. This edition, then, commends itself even more than the preceding, to the profession as an elaborate and complete treatise on the subject. F.

